# The Comparison of Immunomodulatory Properties of Canine and Human Wharton Jelly-Derived Mesenchymal Stromal Cells

**DOI:** 10.3390/ijms25168926

**Published:** 2024-08-16

**Authors:** Anna Burdzinska, Iwona Monika Szopa, Kinga Majchrzak-Kuligowska, Aleksander Roszczyk, Katarzyna Zielniok, Paweł Zep, Filip Andrzej Dąbrowski, Tanushree Bhale, Marek Galanty, Leszek Paczek

**Affiliations:** 1 Department of Physiological Sciences, Institute of Veterinary Medicine, Warsaw University of Life Sciences, Nowoursynowska Str. 159, 02-776 Warsaw, Poland; iwona_szopa@sggw.edu.pl (I.M.S.); kinga_majchrzak@sggw.edu.pl (K.M.-K.); tanushree1999@hotmail.com (T.B.); 2 Department of Clinical Immunology, Medical University of Warsaw, Nowogrodzka Str. 59, 02-006 Warsaw, Polandleszek.paczek@wum.edu.pl (L.P.); 3 Laboratory of Cellular and Genetic Therapies, Center for Preclinical Research, Medical University of Warsaw, Banacha Str. 1B, 02-097 Warsaw, Poland; katarzyna.zielniok@wum.edu.pl; 4 Veterinary Clinic “ochWET”, Pruszkowska Str. 19/21, 02-119 Warsaw, Poland; 5 Department of Gynecology and Gynecological Oncology, Medical Centre of Postgraduate Education CMKP, Marymoncka Str. 99/103, 00-416 Warsaw, Poland; filip.dabrowski@cmkp.edu.pl; 6 Department of Small Animal Diseases and Clinic, Warsaw University of Life Sciences—SGGW, Nowoursynowska 159c, 02-776 Warsaw, Poland; marek_galanty@sggw.edu.pl

**Keywords:** human mesenchymal stromal cells, canine mesenchymal stromal cells, umbilical cord, immunomodulation, transforming growth factor beta, indoleamine 2,3-dioxygenase 1

## Abstract

Although therapies based on mesenchymal stromal cells (MSCs) are being implemented in clinical settings, many aspects regarding these procedures require further optimization. Domestic dogs suffer from numerous immune-mediated diseases similar to those found in humans. This study aimed to assess the immunomodulatory activity of canine (c) Wharton jelly (WJ)-derived MSCs and refer them to human (h) MSCs isolated from the same tissue. Canine MSC(WJ)s appeared to be more prone to in vitro aging than their human counterparts. Both canine and human MSC(WJ)s significantly inhibited the activation as well as proliferation of CD4+ and CD8+ T cells. The treatment with IFNγ significantly upregulated indoleamine-2,3-dioxygenase 1 (IDO1) synthesis in human and canine MSC(WJ)s, and the addition of poly(I:C), TLR3 ligand, synergized this effect in cells from both species. Unstimulated human and canine MSC(WJ)s released TGFβ at the same level (*p* > 0.05). IFNγ significantly increased the secretion of TGFβ in cells from both species (*p* < 0.05); however, this response was significantly stronger in human cells than in canine cells. Although the properties of canine and human MSC(WJ)s differ in detail, cells from both species inhibit the proliferation of activated T cells to a very similar degree and respond to pro-inflammatory stimulation by enhancing their anti-inflammatory activity. These results suggest that testing MSC transplantation in naturally occurring immune-mediated diseases in dogs may have high translational value for human clinical trials.

## 1. Introduction

Over the past decades, epidemiological data suggest evidence of a steady increase in the prevalence of diseases with an autoimmune component [1]. There is an urgent need to improve prevention and develop new therapeutic strategies in this field. One of the methods being tested is the transplantation of mesenchymal stromal cells (MSCs). It has been demonstrated that MSCs possess anti-inflammatory, tolerogenic, and pro-healing activity [2]. The immunomodulatory properties of human MSCs are the subject of extensive research. These cells are generally known to inhibit the activation and proliferation of most lymphocyte subsets while promoting the formation of regulatory T cells (Tregs). The immunosuppressive activity of MSCs is induced/enhanced by a pro-inflammatory environment, which in vitro is simulated by the addition of interferon gamma (IFN-γ), tumor necrosis factor (TNF) or Toll-like receptor (TLR)-3 ligand [3,4]. MSC-mediated immunomodulation is currently believed to be associated with the expression of several molecules, including transforming growth factor beta (TGFβ), indoleamine 2,3-dioxygenase 1 (IDO1), and prostaglandin E2 (PGE2). Cells with MSC characteristics can be relatively easily isolated from different tissues and expanded in vitro while maintaining their therapeutically beneficial properties. This provides the opportunity to test MSCs as an immunomodulatory agent in various pathological conditions. There are already several approved MSC-based therapeutic products worldwide. Some of them are registered for the management of immune-mediated disorders such as graft-versus-host-disease (GVHD) or in perianal Crohn’s disease [5]. It is expected that, in the near future, MSCs can be successfully used in the treatment of other immune-mediated disorders. Nevertheless, there is an agreement that many aspects regarding MSC transplantation procedures require further optimization, as only about 50% of patients with perianal fistulas benefit from MSC administration (based on results published by Panes at al. [6]). At this stage of therapy development, further optimization is usually carried out in experimental animal studies. However, testing the effects of cell-based therapies in rodent models was shown to have rather poor predictive value for clinical outcomes in humans [7]. Another approach that could generate complementary data in translational medicine is to exploit the similarities between diseases affecting humans and companion animals [8,9,10]. As in humans, domestic canines are influenced by environmental factors on a daily basis. These include exposure to stress, a lack of proper diet and physical activity, a low diversity of mucosal microbiota, and air pollution, among others. Not surprisingly, companion dogs suffer from many diseases that are very similar to those occurring in humans. A number of these disorders result from the dysregulation of the immune system [8]. For example, dogs develop chronic enteropathy (sharing similarities with inflammatory bowel disease in humans), perianal furunculosis (considered a model of anal fistulas in Crohn’s disease), dry conjunctivitis and keratitis (considered a model of Sjögren’s syndrome), atopic dermatitis, pemphigus deciduous, lupus erythematosus, as well as type I diabetes [8,10,11]. Additionally, dogs suffer from malignant and nonmalignant blood disorders. Although hematopoietic cell transplantation is still rare in veterinary practice, it is developing. This in turn may be related with increased prevalence of GVHD in dogs [12]. It is believed that the knowledge resulting from testing MSC transplantation in dogs can have significant therapeutic potential in human clinical trials while leading to the development of new therapies in veterinary medicine.

To successfully conduct such translational studies, it is necessary to define the properties of canine mesenchymal stromal cells and compare them to human counterparts. It was already shown that canine MSCs display a similar phenotype and differentiation capacity to human MSCs [13], but there are far less data regarding their immunomodulatory properties [10,14]. Therefore, the aim of this study was to explore the immunomodulatory properties of canine MSCs while assessing the translational value to human counterparts isolated from the same tissue.

## 2. Results

### 2.1. Isolation of Human and Canine Umbilical Cord-Derived Cells and Their Growth in Standard Conditions

**Figure 1:**
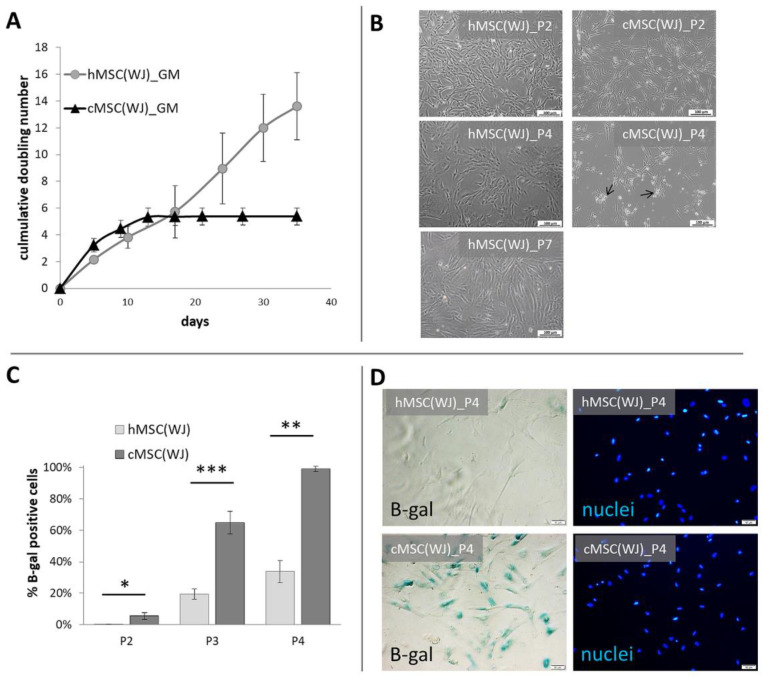
Growth dynamics and senescence of human and canine umbilical cord-derived MSCs in standard conditions. (**A**) Cumulative doubling number of representative human (h) and canine (cMSC(WJ)) cells which were cultured for 35 days (day 0—1st passage), (mean values, SD, *n* = 3). (**B**) The appearance of human (left column) and canine (right column) cells in standard conditions after the 2nd (P2, upper row),4th (P4, middle row), and 7th (P7, lower picture, human only) passage. Black arrows indicate fragmented cells, scale bars–100 μm (**C**) The mean (±SEM) proportion of cells displaying senescence associated β-galactosidase (β-gal) activity in human and canine populations on subsequent passages (*p*). *, *p* < 0.05; **, *p* < 0.01; ***, *p* < 0.001, Student’s *t*-test, *n* = 5. (**D**) Assessment of cell senescence using microscopy: images in rows present the same fields of view. Left column—cells with β-gal activity are blue, right column—cell nuclei stained in blue, scale bars–50 μm.

Within the first days of the culture, cells with a mesenchymal appearance migrated out of the cut-to-piece human umbilical cord matrix. Human WJ-derived fibroblastic cells proliferated on an uncoated surface for at least seven passages (35 days) without noticeable morphological changes (Figure 1A,B). Canine perinatal tissue also gave rise to cells with similar morphology within the first few days of culture. Cells cultured in conditions used for human cells (in an uncoated surface with standard GM) proliferated intensively until the second passage, where they were observed to slow down. After the fourth passage, the cells ceased to divide while also displaying a changed morphology—cells were large, flattened, often fragmented (Figure 1A,B). After excluding microbial contamination of the culture (including Mycoplasma spp.) as a potential cause, the cells were examined for senescence. Both human and canine cells cultured in standard conditions (GM) were tested after three subsequent passages (the second, third, and fourth). The percentage of SA-β-galactosidase positive cells was also calculated. We witnessed that the number of senescent cells increased in cells from both species, but canine cells displayed a significantly higher proportion of aged cells than their human counterparts at each passage (*p* < 0.05). After the fourth passage, over 95% of canine cells displayed the activity of SA-β-gal in comparison to 25% in human populations (Figure 1C,D).

### 2.2. Optimization of Culture Conditions for Canine WJ-Derived Cells

**Figure 2:**
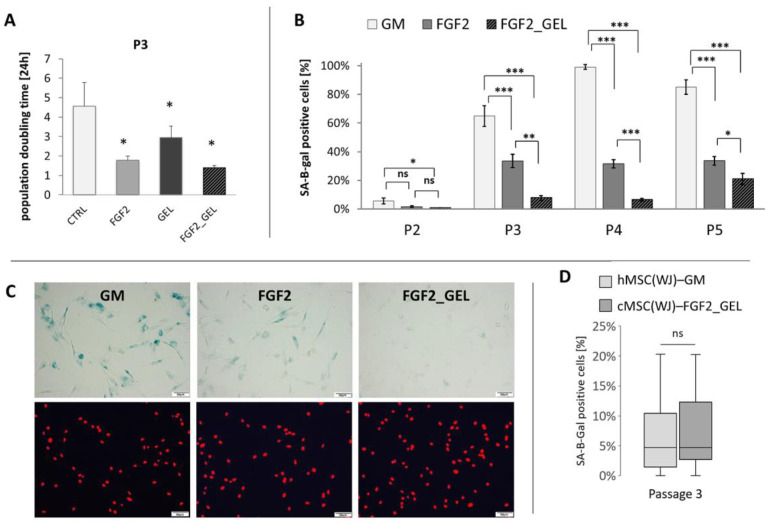
The effect of different culture conditions on population doubling time (PDT) and senescence of canine Wharton jelly-derived mesenchymal stromal cells, MSC(WJ). (**A**) PDT of canine MSC(WJ) cultured in growth medium (GM), GM with 2.5 ng/mL FGF2 (FGF2), in GM on gelatin-coated surface (GEL), and GM + FGF2 on gelatin-coated surface (FGF2_GEL), *n* = 6, *—*p* < 0.05 in comparison to control using Wilcoxon test or Student’s *t*-test for related data test depending on data distribution; (**B**) The proportion of senescent cells within the population (mean ±SEM) cultured in GM, FGF, and FGF2_GEL in subsequent passages (*p*). Data analyzed within each passage using one-way ANOVA with post hoc Tukey’s test. *, *p* < 0.05; **, *p* < 0.01; ***, *p* < 0.001; *n* ≥ 5; (**C**) representative images used for calculation β-gal assay (using X-gal substrate for reaction). Images in columns represent the same field of view. Upper row—light microscopy, senescent cells visible as blue, lower row—fluorescent microscopy, cell nuclei stained in red. Scale bars—50 μm. (**D**) The comparison of cell senescence at third passage (P3) between human MSC(WJ)s cultured in GM on uncoated surface and canine MSC(WJ) cultured in FGF2_GEL conditions. The graph presents median, quartiles, min and max values, *n* = 8. Data analyzed using Mann–Whitney U assay. ns—*p* > 0.05.

The rapid in vitro aging of canine WJ-derived cells revealed a need to optimize the culture conditions in order to produce the sufficient number of cells for potential transplantation without significant signs of cellular senescence. The following four different culture conditions were tested: 1. cells on an uncoated surface in GM (CTRL), 2. cells on an uncoated surface in GM supplemented with 2.5 ng/mL FGF2 (FGF2), 3. cells in GM cultured on a gelatin-coated surface (GEL), 4. Cells on a gelatin-coated surface in GM with 2.5 ng/mL FGF2 (GEL + FGF2). All tested modifications of culture conditions significantly increased the metabolic activity (referred also as viability) of the cWJ-derived populations in comparison to control. After 48 h of culture, the mean viability of cells in the FGF2, GEL and FGF2_GEL groups was 1.9, 1.5, and 2.2-fold higher than in the CTRL group, respectively (Supplementary Figure S1A). Observed increase in metabolic activity resulted partially from enhanced proliferation, which was confirmed by the assessment of population doubling time calculated between the second and fourth passage. The mean PDT of canine UC-derived cells from the control group amounted to 4.5 days, whereas the mean PDT in the FGF2, GEL, and FGF2_GEL groups amounted to 1.8, 2.9, and 1.4 days, respectively (*p* < 0.05 for all conditions in comparison to control, Figure 2A). Similarly, the proportion of dead cells in collected populations was lower in groups with modified conditions than in the CTRL group. For the FGF2_GEL group, the difference was statistically significant (Supplementary Figure S1B).

The analysis of cell senescence revealed that addition of FGF2 significantly reduced the proportion of aged cells in canine populations, and the addition of FGF2 with a gelatin coating further significantly decreased cell senescence (Figure 2B,C). The maintenance of canine MSC(WJ)s under these conditions (FGF2_GEL) resulted in a population at the fourth passage in which 6.5% of the cells showed signs of senescence, while the same cells cultured in control medium showed 99% senescence, *p* < 0.001. The percentage of senescent canine cells cultured in FGF2_GEL conditions was not significantly different than the proportion of senescent human MSC(WJ)s cultured in standard conditions, i.e., in GM on an uncoated surface (Figure 2D).

### 2.3. Identification of Human and Canine UC-Derived Cells

**Figure 3:**
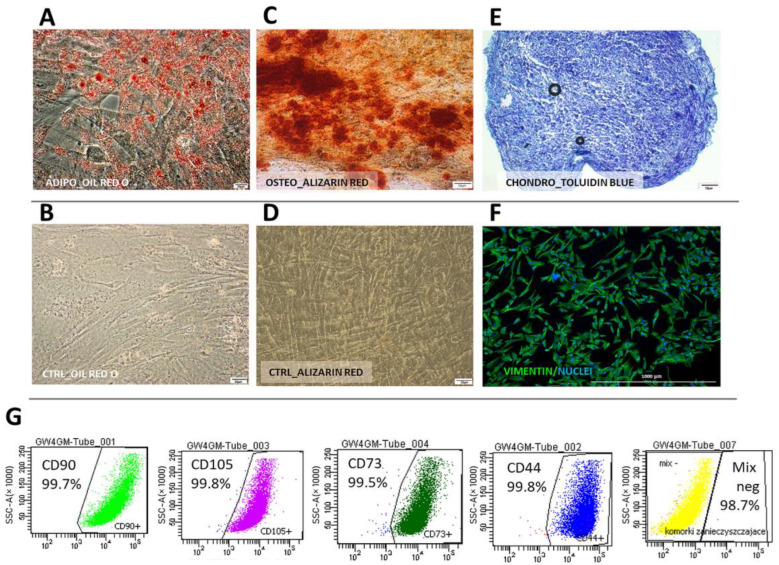
Human Wharton jelly-derived mesenchymal stromal cells (cMSC(WJ)s) identification. (**A**–**F**) differentiation. Oil Red O-stained hMSC(WJ)s cultured in (**A**) adipogenic medium. Lipid droplets stained in red. and (**B**) in standard growth medium (CTRL); Alizarin Red stained hMSC(WJ)s cultured in (**C**) osteogenic medium. Calcium deposits stained in red and (**D**) in GM; (**E**) chondrogenic differentiation, toluidine blue staining. Proteoglycans stained in purple; (**F**) immunocytochemistry. hMSC(WJ) stained for the presence of vimentin intermediate filament expressed in mesenchymal cells (**G**) Flow cytometry analysis of surface antigens: cells are positive for CD90, CD44, CD73, CD105. Negative cocktail (MIX neg) consisted of CD34, CD45, CD11b, CD19 and HLA-DR. Individual plots from representative population, Scale bars: 20 µm (**A**,**B**); 50 µm (**C**–**E**); 1000 μm (**F**).

**Figure 4:**
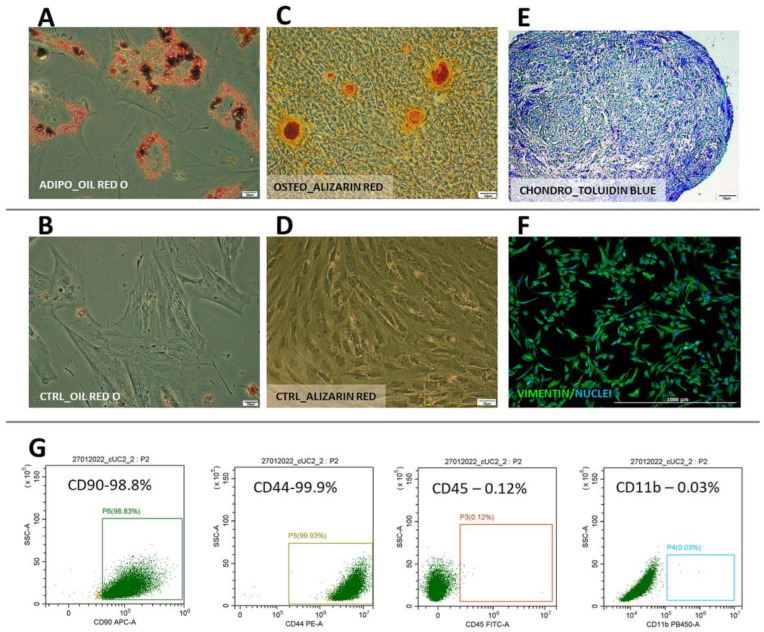
Canine Wharton jelly-derived mesenchymal stromal cells (cMSC(WJ)s) identification. (**A**–**E**) differentiation. Oil Red O-stained cMSC(WJ)s cultured (**A**) in adipogenic medium, lipid droplets are red and (**B**) in standard growth medium (CTRL); Alizarin Red stained cMSC(WJ)s cultured (**C**) in osteogenic medium, calcium deposits are red and (**D**) in GM; (**E**) chondrogenic differentiation, toluidine blue staining, proteoglycans stained in purple; (**F**) immunocytochemistry. cMSC(WJ) stained for the presence of vimentin intermediate filament expressed in mesenchymal cells; (**G**) Flow cytometry analysis of surface antigens: cells are positive for CD90, CD44, negative for CD11b, CD45; Scale bars: 20 µm (**A**,**B**); 50 µm (**C**–**E**); 1000 µm (**F**).

Cells from both species displayed typical mesenchymal differentiation potential, which was confirmed by successful differentiation into adipogenic, chondrogenic, and osteogenic lineages (Figure 3A–E and Figure 4A–E). In human WJ-derived populations, the cells expressed CD90 (98.7 ± 0.42%), CD105 (98.6 ± 0.4%), CD73 (98.8 ± 0.32), CD44 (98.8 ± 0.4%) and did not have CD11, CD19, CD34, CD45, HLA-DR (98.8 ± 0.7%) on their surface (Figure 3) thus meeting the minimal criteria for mesenchymal stromal cells [15]. Canine cells expressed CD90 (99.8 ± 0.03%), CD44 (99.5 ± 1.5%) and did not have CD45 (99.9 ± 0.03%) and CD11b (99.9 ± 0.01%) on their surface (Figure 4). Moreover, all cells within both the human and canine types expressed vimentin intermediate filaments typical of mesenchymal lineages.

### 2.4. The Effect of Human and Canine MSC(WJ)s on the Proliferation of T Cells

**Figure 5:**
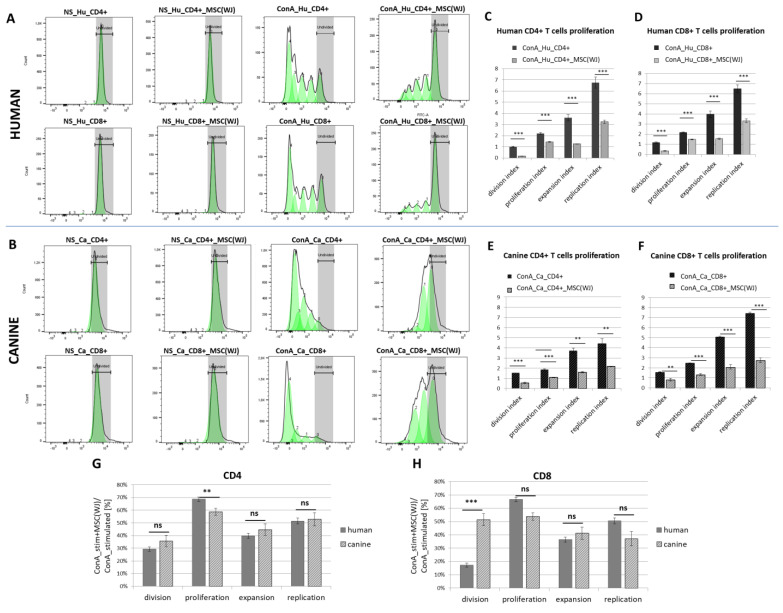
The effect of Wharton jelly-derived mesenchymal stromal cells (MSC(WJ)s) on T cell proliferation. Dye dilution assay assessed using flow cytometry (CellTrace™). Representative histograms of human (**A**) and canine (**B**) T cells (CD3+) stained with membrane fluorochrome. Upper rows represent CD4+ T cells, lower rows—CD8+ T cells. Columns from the left present: (1) non-stimulated (NS) T cells (fully stained, the region were undivided cells locate is marked in gray), (2) non-stimulated T cells co-cultured with species-matched MSC(WJ), (3) concanavalin A (ConA) stimulated T cells—subsequent generations of lymphocytes have more and more diluted dye on their surface, (4) ConA stimulated T cells with addition of species-matched MSC(WJ); populations after subsequent divisions are marked as green peaks; (**C**–**F**) Graphs present mean (±SEM) values of proliferation associated indexes. Graphs show mean (±SEM) values of proliferation-related indices for human CD4+ (**C**), human CD8+ (**D**), canine CD4+ (**E**), and canine CD8+ (**F**). Each data pair shows the index for cells stimulated with ConA (black bars) and the same cells stimulated with the addition of species-compatible MSC(WJ)s (gray bars). **, *p* < 0.01; ***, *p* < 0.001, ns—*p* > 0.05; *n* = 6–8, MSC(WJ)s from four different donors (for each species) were used for experiments. (**G**,**H**) comparison of the effect of human and canine MSC(WJ)s on the proliferation of T cells.

Species-matched PBMCs and MSC(WJ) were co-cultured for 5 days. Neither canine nor human MSC(WJ)s stimulated the proliferation of untreated lymphocytes from unrelated donors in any case (Figure 5A,B, second column). Concanavalin A potently stimulated the proliferation of human and canine lymphocytes (both CD4+ and CD8+ subsets). The addition of MSCs to PBMCs (ratio 1:10) significantly inhibited both activation and proliferation of CD4+ and CD8+ T cells, regardless of MSC species origin (Figure 5A,B). The mean values of all calculated indexes were significantly lower in the presence of MSCs compared to cultures without MSCs (*p* < 0.01 in all cases, Figure 5C–F). In the next step, the indexes after adding MSC were calculated as a percentage of the control value (ConA stimulated + MSC(WJ)/ConA stimulated × 100 [%]), and then the analogous values obtained for human and canine cells were compared statistically. This analysis revealed that CD4+ T cell proliferation index decreased significantly more after the addition of canine MSC(WJ)s than human MSC(WJ)s to species-matched activated lymphocytes (*p* < 0.01). In CD8+ T cells, the decrease in division index driven by MSC(WJ)s was significantly more profound in human cells than in canine cells (*p* < 0.001). Other comparisons showed no differences between the effects of human and canine MSC(WJ)s on species-matched lymphocytes (Figure 5G,H).

### 2.5. The Effect of Pro-Inflammatory Treatment on the Immunomodulatory Properties of Human and Canine MSC(WJ)s

#### 2.5.1. The Effect of Pro-Inflammatory Treatment on IDO1 Protein Synthesis

**Figure 6:**
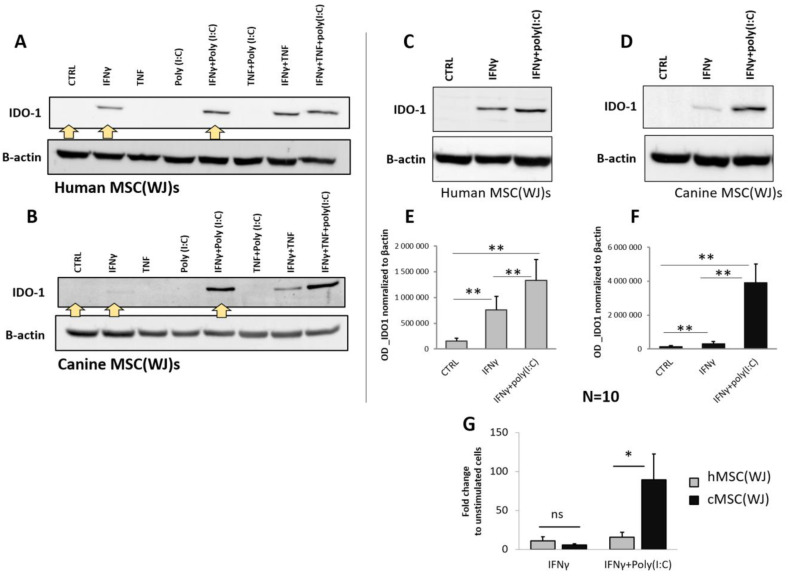
The effect of pro-inflammatory stimulation on the synthesis of IDO1 in human and canine MSC(WJ)s. Western blot. (**A**,**B**) Representative blots presenting IDO1 protein expression in human (**A**) and canine (**B**) MSC(WJ)s treated for 24 h with different combinations of IFNγ, TNF, and poly(I:C). Yellow arrows indicate treatments chosen for extended analysis. (**C**–**G**) Representative blots presenting the effect of chosen treatments: IFNγ and IFNγ + poly(I:C) in human (**C**) and canine (**D**) MSC(WJ)s. (**E**,**F**) Mean (±SEM) optical density of IDO-1 normalized to β-actin (*n* = 10) in hMSC(WJ)s (**E**) and cMSC(WJ)s (**F**); 3 independent experiments, cells from 6 different donors in each species, *n* = 10, analyzed using Wilcoxon test, **, *p* < 0.01. (**G**) the comparison of the effect of both treatments on hMSC(WJ)s and cMSC(WJ)s. Mean (±SEM) fold change after treatment in comparison to untreated cells. Mann–Whitney U test, ns—statistically non-significant, *, *p* < 0.05.

For the selection of pro-inflammatory treatment, we assessed the synthesis of IDO1. By synthesis, we mean protein production/expression, which was analyzed in cell lysates using Western blot. In the preliminary phase, cells were treated with all possible combinations of the following three pro-inflammatory stimuli: IFNγ (20 ng/mL), TNF (20 ng/mL), and poly(I:C) (5 µg/mL). This resulted in seven different treatments and a control. Both human and canine cells cultured in control medium showed no visible band corresponding to the IDO1 protein in WB analysis. When the agents were used individually, only IFNγ induced a visible increase in IDO1 production, while TNF and poly(I:C) did not have such an effect. When the agents were used in combinations, only those containing IFNγ induced an increase in IDO1 protein synthesis (Figure 6A,B). The effect of the combination seemed to be additive in nature, which was particularly evident in the treatment of IFNγ + poly(I:C) in canine cells. To confirm this, we further treated the cells with IFNγ and the combination of IFNγ + poly(I:C) using cells from six different donors in each species. Stimulation of MSC(WJ)s with IFNγ (20 ng/mL, 24 h) resulted in a mean 11-fold (±5.0) increase in IDO1 production in human cells and 5.5-fold (±1.7) in canine cells (*p* < 0.05 compared to controls for cells from both species). Treatment of MSC(WJ)s with IFNγ + poly(I:C) resulted in a 15-fold (±6) increase in IDO-1 synthesis (vs. CTRL) in human MSC(WJ)s and an 89-fold (±33) increase in canine cells (both *p* < 0.01). Moreover, cells treated with IFNγ + poly(I:C) produced significantly more IDO1 than cells treated with IFNγ alone (*p* < 0.05 for cells from both species, Figure 6E,F). A direct comparison of the response of human and canine MCS(WJ)s to treatment showed that the response to IFNγ (in terms of IDO1 synthesis) was the same regardless of the species origin of cells, while the response to treatment with the IFNγ + poly(I:C) combination was significantly stronger in the case of canine cells than in human cells (*p* < 0.01, Figure 6G).

#### 2.5.2. The Effect of Pro-Inflammatory Treatment on TGFβ1 Secretion

**Figure 7:**
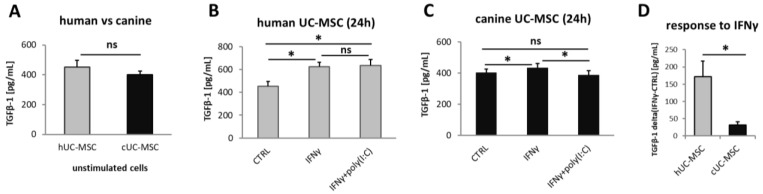
Transforming growth factor beta 1 (TGFβ1) secretion by MSC(WJ)s. ELISA. Data blanked to medium alone. (**A**) basal secretion of TGFβ1 by unstimulated human and canine MSC(WJ)s. (**B**,**C**) the effect of pro-inflammatory stimulation on TGFβ1 secretion by human (**B**) and canine (**C**) cells. (**D**) comparison of response to recombinant (species-matched) IFNγ (20 ng/mL) on TGFβ1 secretion between human and canine MSC(WJ)s. The graph presents mean delta values (TGFβ1IFNγ—TGFβ1CTRL). ns—statistically not significant, *, *p* < 0.05; *n* = 6 for each cell type, assay performed in duplicates.

The TGFβ1 was secreted by unstimulated hMSC(WJ)s and cMSC(WJ)s in all tested populations (*n* = 6 for each cell type). The medium alone contained no TGFβ1. The mean concentration of TGFβ1 in supernatants collected from untreated hMSC(WJ)s and cMSC(WJ)s amounted to 452 pg/mL/24 h and 401 pg/mL/24 h, respectively, and did not differ statistically from each other (*p* > 0.05, Figure 7A). The treatment of MSC(WJ)s with IFNγ (20 ng/mL) resulted in a significant increase in TGFβ1 secretion in both human and canine cells in comparison to untreated controls (*p* < 0.05, Figure 7B,C); however, this response was significantly more pronounced in human cells than in canine cells (*p* < 0.05, Figure 7D). The treatment of MSC(WJ)s with the IFNγ + poly(I:C) combination gave more variable results. In human cells, the response did not differ from the effect of IFNγ alone, but in canine cells, the addition of poly(I:C) to IFNγ reduced the effect of IFNγ—the average secretion of TGFβ1 was significantly lower than after treatment with IFNγ and did not differ from CTRL (Figure 7B,C).

## 3. Discussion

The aim of this study was to assess the immunomodulatory properties of canine MSC(WJ)s and compare them to human counterparts. There are three most commonly used sources of MSCs in human clinical trials—bone marrow (BM), adipose tissue (AT) and umbilical cord matrix also called Wharton’s jelly [16]. In the present study, we used an umbilical cord matrix as a source of MSCs. This decision resulted from the fact that human MSCs with a fetal origin tend to have better proliferative potential than MSCs from the BM-derived counterparts [17]. Moreover, perinatal tissues are easily accessible in large amounts and, finally, it was shown many times (also by our group) that, in terms of the ability to inhibit lymphocyte proliferation, hMSC(WJ)s are not inferior to human MSCs from other sources (such as adipose tissue or bone marrow) [18,19,20].

Before assessing the immunomodulatory activity, it was necessary to obtain healthy, able to proliferate cells at the level of passage 3–4 (usually, such cells are used for transplantation in pre-clinical and clinical trials). Human MSC(WJ)s are well known to display stable growth in standard conditions (DMEM + 10% FBS) without changing morphology and functionality for several passages [18,19], which was confirmed in the present study. However, we noticed that canine MSC(WJ)s in these conditions slowed down the proliferation rate already after the third passage. This observation was in agreement with previously published results [21,22,23]. It was reported that canine umbilical cord matrix-derived MSCs stop growing on early passages in contrast to the canine placenta-derived MSCs [21]. Another group demonstrated that adding FGF2 (10 ng/mL) to the growth medium and covering the culture surface with gelatin significantly enhanced canine MSC(WJ) longevity [23]. We confirmed the results of Wright at al. and, additionally, we demonstrated (using SA-β-gal assay) that the observed growth arrest in the conditions used for culturing human cells is the result of the massive premature in vitro senescence. Because this aging process took place at such an early stage of cultivation, it was clear that it was not due to intensive multiplication and the reaching of the so-called Hayflick limit [24]. We have shown that canine MSC(WJ)s cultured in DMEM + 10% FBS with 2.5 ng/mL FGF2 on gelatin-coated dishes undergo more divisions and, at the same time, display a significantly lower level of senescence than the same cells cultured in DMEM + 10% FBS only. Our data, combined with the results of other groups, clearly indicate that canine MSC(WJ)s have different growth requirements than human MSC(WJ)s. It appears that canine MSC(WJ)s need stronger (than human cells) signals from growth factors and adhesion molecules to maintain their ability to proliferate in vitro. In the absence of the appropriate stimulation, an aging process is activated in this population. The need to use different culture conditions for canine and human cells during the growth phase poses certain limitation in our comparative study. These observations are critical in planning potential clinical trials in dogs using MSCs of umbilical cord matrix origin.

In the next step, the cells underwent identification assays. For human cells, the generally accepted minimal criteria for MSCs identification were set out in the International Society for Cell and Gene Therapy position statement [15]. The cells used in the present study met all the expected criteria. In the case of animal cells, the identification criteria have not been so formally defined. However, in 2022, Guest et al. proposed the minimal criteria for identifying MSCs in veterinary research, in particular for canine and equine MSCs [25]. The authors pointed out the need to demonstrate the adhesion to plastic, the presence of at least two positive markers and the absence of at least two negative markers, and the ability to undergo three-lineage differentiation. To ensure that the modified culture conditions did not affect the basic characteristics of canine MSCs, the identification study was performed on cells that had been cultured on gelatin with the addition of FGF2 since isolation. The cells met all minimal criteria which indicates that the modified culture conditions enabled effective multiplication of canine MSCs(WJ) in vitro without changing their basic characteristics.

It is believed that a key immunomodulatory feature of MSCs is their influence on the activity of lymphocytes. Katarina Le Blanc and colleagues were the first who showed that MSCs added to stimulated lymphocytes significantly inhibit their activation and proliferation [26]. This observation, with respect to human MSCs from various sources, has been repeatedly confirmed by other researchers, including our group [2,18,19]. However, data regarding immunomodulatory properties of canine MSCs are still scarce [14,27]. As our goal was not only to assess the effects of cMSC(WJ)s on T cells but also to compare this feature between canine and human MSC(WJ)s, we aimed to use a maximally unified protocol for cells from both species. Therefore, to activate T cells, we chose concanavalin A—a well-known plant-derived lymphocyte mitogen instead of allostimulation or species-specific CD3/CD25 activators. Members of our group have previously demonstrated that ConA can be successfully used for the activation of both CD4+ and CD8+ canine T cell subsets [28]. In the present study, we demonstrated that canine MSC(WJ)s added to canine PBMCs from unrelated, healthy donors significantly suppressed the activity of both CD4+ and CD8+ T cells. For an extended analysis, a proliferating cell model fitting can be implemented using dedicated software as described [29]. This type of analysis was also confirmed to be an effective way to assess the influence of MSCs on T cell proliferation [30]. Herein, we have demonstrated that adding MSCs (regardless of species origin) to our experimental setup (1:10 ratio of MSC:PBMC, stimulation with ConA, 4 days co-culture with MSCs) significantly reduced all calculated indexes related to T cell activity (division, proliferation, expansion, and replication indexes) in comparison to control (stimulated lymphocytes without species-matched MSCs). In addition, we compared the extent of this inhibition between human and canine cells. Out of eight compared parameters (four indexes in each T cell subset: CD4+ and CD8+), we observed statistically significant difference between the impact of human and canine MSCs on T cells in only two cases. Human MSC(WJ)s appeared to be more effective at inhibiting CD8+ T cell activation than canine MSC(WJ)s (based on the significant difference in the effect on the division index between the two analyzed cell types). On the other hand, canine MSC(WJ)s had a stronger inhibitory effect on CD4+ proliferation than human MSC(WJ)s (based on the significant difference in the effect on the proliferation index between human and canine cells). As we studied a dynamic co-culture system, it is difficult to judge whether the differences result from the various activities of the MSCs or immune cells. Regarding the replication and expansion indexes, no differences in the effect of MSCs on T cells were noted between human and canine cells. Our results clearly demonstrate that canine MSC(WJ)s can suppress the activity of stimulated T cells from unrelated, healthy donors. The extent of this inhibition is very similar to that noted in human counterparts. The observed minor differences in canine and human MSCs-T cells interactions may be an area for further research.

It is postulated that one of key factors mediating interactions between MSCs and immune cells is indoleamine-2,3-dioxygenase 1 [4,31]. IDO1 is an enzyme that breaks down tryptophan into kynurenine. Both tryptophan depletion and kynurenine accumulation result in T cell inhibition and Treg cell induction. It was previously shown that blocking IDO1 activity significantly reduces the ability of MSCs to suppress the proliferation of lymphocytes [4]. Our results indicate that IDO1 protein expression in unstimulated MSCs is barely detectable by Western blot, which is in line with previously published data [4,31]. It is known from numerous previous studies that IFNγ is a potent inducer of IDO1 expression in many cell types, and that this induction mainly depends on STAT1 and IRF1 transcription factors [32]. The same response to IFNγ in terms of IDO1 expression was previously shown in human MSCs, regardless of their tissue of origin [31]. Our data confirm this observation in human MSC(WJ)s and additionally show that canine MSC(WJ)s also respond to IFNγ with a significant increase in IDO1 production (*p* < 0.01) and that the intensity of this response (compared to unstimulated control) does not differ between human and canine MSC(WJ)s. Our results further demonstrate that both TNF and TLR3 ligand act synergistically with IFNγ on IDO1 protein expression in MSCs and that the general pattern of response to various combinations of these factors is the same in human and canine cells. Our data clearly indicate that the main inducer of IDO1 is IFNγ as the two other factors added alone did not increase IDO1 synthesis, but they did enhance the response to IFNγ when used in combination with the latter. The additive effect of IFNγ with TNF on MSCs was recently demonstrated in terms of PD-L1 expression by Chen at al. (2023) [33]. The authors proposed that the mechanism of enhancing the effect of IFNγ by TNF is through upregulating the expression of IFNGR1/2 via the NF-κB pathway, which promotes the activation of the JAK/STAT1/IRF1 pathway induced by IFNγ. It is very likely that the synergistic effect of IFNγ and TNF in relation to the IDO1 expression observed in our study is based on the same intracellular interactions, but this hypothesis would require further research. TLR3 ligand is the next factor which has been postulated to be an enhancer of the immunomodulatory properties of MSCs; however, the previous data in regard to its effect on IDO1 expression in MSCs are varied. Opitz and colleagues demonstrated that stimulation of hBM-MSCs with poly(I:C) (50 μg/mL) alone increased significantly the expression of IDO1 [4]. On the other hand, it was reported that stimulation of hMSC(WJ)s with 1 μg/mL of poly(I:C) upregulated PGE2, but not IDO1 [34]. The difference between these observations may be due to the significant discrepancy in the poly(I:C) doses used (50 μg/mL vs. 1 μg/mL), but Kim and colleagues [31], using 100 ug/mL of poli(I:C), also observed no increase in IDO1 expression in human MSCs regardless of their tissue origin while observing a strong response to IFNγ. Our results are in line with data of the latter mentioned group, but we have demonstrated that poli(I:C) synergize the effect of IFNγ on IDO1 expression. Such an effect was previously reported in murine MSCs [35]. Importantly, the same group later demonstrated that the priming of murine MSCs with the IFNγ and poly(I:C) improved the therapeutic effect of MSC transplantation in mice with induced colitis. The authors claimed that promoting IDO1 expression had a key role in this effect [36]. Our report showing the same additive effect in human cells gives hope that preconditioning MSCs with IFNγ/poly(I:C) combination may be an attractive method to enhance the immunosuppressive effect of MSCs in clinical trials. Moreover, the demonstration of the same reaction in canine cells allows us to believe that testing the transplantation of preconditioned MSCs in dogs with naturally occurring autoimmune diseases may provide a high-quality prediction for similar therapies in humans.

We have additionally evaluated the secretion of TGFβ1 in analyzed cell types. TGFβ is another factor indicated as key mediator of the immunomodulatory effect of MSCs [37]. It is a well-known immunosuppressive cytokine, inhibiting the proliferation of lymphocytes while promoting T regulatory cells [38]. In contrast to IDO1, the expression and secretion of TGFβ by MSCs does not require the presence of an inflammatory environment. MSCs release significant amounts of this cytokine under standard culture conditions. However, we have previously reported, that the level of TGFβ production by unstimulated human MSCs varies depending on the origin of the MSCs. Moreover, our data indicated that MSC(WJ)s constitute a rich source of this factor [39]. This finding provided an additional argument for choosing UC for MSC isolation in the present study. Herein, we have demonstrated that the basic TGFβ1 secretion (pg/mL/24 h) level does not differ between human and canine MSC(WJ)s. Moreover, cells from both species respond to IFNγ with a statistically significant increase in TGFβ release, although the response of human cells was significantly more pronounced compared to canine cells.

It should be noted that IDO-1 and TGFβ, although very important, are not the only factors associated with the immunomodulatory effect of MSCs. These compounds were selected for analysis because they have well-documented significance in the MSC–T cell interaction. Another factor that could be included in the analysis is PGE2—a potent immunomodulatory factor secreted by MSCs [27,40].

It seems worth emphasizing that MSC(WJ) isolated from dogs and humans had different culture requirements. Although the analyses of immunomodulatory properties were performed under as similar conditions as possible for cells from both species, they were exposed to different conditions during the growth phase. It is possible that these modified conditions could have influenced the behavior of the cells. Additionally, future studies may consider conducting experiments in FBS-free media to obtain data that can be better compared with other studies [41].

Altogether, our results demonstrate that although canine and human MSC(WJ)s have different growth requirements, both populations show the expected immunomodulatory properties. Although these properties differ in detail, cells from both species inhibit the proliferation of activated T cells to a very similar extent and respond to pro-inflammatory stimulation by enhancing their anti-inflammatory activity. These results suggest that testing MSC transplantation in naturally occurring immune-mediated diseases in dogs may have high translational value for human clinical trials. Additionally, we hope our work may contribute to further understanding of canine MSC biology, which may help define species-specific quality standards and assist in the design of appropriate veterinary clinical trials to further verify the clinical efficacy of MSC administration in various diseases.

## 4. Materials and Methods

### 4.1. MSCs Isolation

#### 4.1.1. Human Wharton Jelly-Derived Mesenchymal Stromal Cells (hMSC(WJ)s)

Umbilical cord fragments of approximately 3–5 cm in length were obtained from 6 patients after a planned delivery by caesarean section, with the approval of the Local Bioethics Committee (approval number: KB/32/2018) and after obtaining individual informed consents. The tissue was placed aseptically in sterile phosphate-buffered (PBS) solution with 1% antibiotic solution (Penicillin-Streptomycin, Biowest, Bradenton, FL, USA) and stored at 4 °C until isolation. The isolation process has been initiated maximally 12 h after tissue collection and based on spontaneous migration of fibroblastic cells from WJ explants cut into small pieces, as we described previously [18]. The culture was run in the following conditions: 37 °C, 95% humidity, 5% CO_2_, atmospheric O_2_ in growth medium (GM) consisted of DMEM (low glucose, Biowest) supplemented with 10% fetal bovine serum (FBS, EuroClone, Cat.# ECS5000L) and 1% of antibiotic solution (Penicillin-Streptomycin) on Primaria™ dishes (Corning^®^, Glendale, AZ, USA).

#### 4.1.2. Canine Wharton Jelly-Derived Mesenchymal Stromal Cells (cMSC(WJ)s)

Canine perinatal tissues (fetal membranes and umbilical cords) were obtained during the planned deliveries by caesarean sections performed in companion dogs. Collection of perinatal tissues was carried out in a way that did not affect the well-being of the newborn puppies. The tissue was placed aseptically in (PBS) solution with 1% antibiotic solution and stored at 4 °C until isolation. The Local Ethical Committee approved using this material for research purposes. Perinatal tissues from 5 different litters (in total, they included 12 puppies) were used in this study. Cells isolated from the first two litters were used for optimization of the culture conditions. Immunomodulatory properties were tested on cells from 3 consecutive litters. Tissue from each pup was processed separately. In the cell culture laboratory, the fetal membranes were separated from umbilical blood vessels surrounded by connective tissue which was the source of fibroblastic cells. The latter were cut into pieces and left in culture in the same way as human explants. Therefore, the isolation of cells from both species (human and canine) was based on spontaneous migration of fibroblastic cells from WJ tissue. In the first phase of this study, canine tissues were kept in the same physical conditions and basal growth medium as human counterparts. After optimization process, cMSC(WJ)s were cultured on gelatin-coated dishes in GM supplemented with 2.5 ng/mL of fibroblast growth factor (FGF2) through the entire period of culture (from isolation to the beginning of experiments evaluating immunomodulatory properties).

### 4.2. Cell Culture

Cells were passaged whenever the culture reached approximately 80–90% confluence. After the second or the third passage, the cells were cryopreserved (DMEM/50% FCS/10%DMSO). Some vials were thawed for identification procedures. The remaining cells were recovered successively for the use in experiments described below. Cells isolated from different donors (within a litter each pup considered as different donor) were always cultured separately (not pooled). Different pups within one litter were considered to be separate donors. Cells from at least three different donors (for each species) were used for each type of experiment in this study. Additionally, in the case of canine cells, MSCs from at least two different litters were used in each experiment. All experiments (except experiments for optimizing culture conditions) were run on cells at the fourth or the fifth passage.

### 4.3. Viability Assays

For the cell viability assay, cells were seeded on a 96-well plate in a density of 8 × 10^3^/well. On the day of analysis, MTT dye (3-(4,5-dimethylthiazol-2-yl)-2,5-diphenyltetrazolium bromide) was added to a culture (to obtain final concentration—1 mg/mL). After 2 h under standard culture conditions, the medium was discarded, and the formed formazan crystals were dissolved with dimethyl sulfoxide. Samples were analyzed colorimetrically at a wavelength of 570 nm. All tests were performed in triplicates. At least six biological replicates were used in each group.

### 4.4. Population Doubling Time (PDT) Calculation

To assess proliferation rate, cells were seeded on Primaria™ dishes at a density of 5 × 10^3^/cm^2^. After five days, cells were collected and stained with Trypan blue. The number of both the viable and dead cells was counted manually using a hematocytometer. Doubling time was assessed using the following online calculator: https://www.doubling-time.com/compute.php, accessed on 20 January 2022.

### 4.5. In Vitro Senescence Assessment (β-Galactosidase Assay)

As canine cells significantly inhibited their growth after the third passage (despite being free of *Mycoplasma* spp. (verifed using PCR) and other aerobic microorganisms (verified by culture in microbiology laboratory), the in vitro aging process was evaluated in the cultured cells using a protocol described by Debacq-Chainiaux et al. [42]. To assess the activity of the senescence-associated (SA) β-galactosidase, cells were treated with fixative solution (2% (*w*/*v*) paraformaldehyde, 0.2% (*v*/*v*) glutardehyde in phosphate buffered solution (PBS)) for 5 min, washed, and then treated with X-gal staining solution (40 mM citric acid/Na phosphate buffer, 5 mM K_4_[Fe(CN)_6_] 3H_2_O, 5 mM K_3_[Fe(CN)_6_], 150 mM sodium chloride, 2 mM magnesium chloride and 1 mg/mL X-gal in distilled water). X-gal is the substrate of the reaction catalyzed by SA-β-gal. The product of this reaction is blue, which allows for the identification of cells undergoing the aging process. After administration of the X-gal solution, cells were incubated overnight (12–16 h) in 37 °C (in atmospheric oxygen—21% O_2_). After washing, cell nuclei were counterstained with one of the fluorescent dyes—4′,6-diamidyno-2-fenyloindol (DAPI) or 7-aminoactinomycin D (7AAD). Cells in each well were documented by imaging in both the bright field (to visualize blue product of enzymatic reaction) and fluorescent modes to visualize cell nuclei. The images were analyzed using cellSens software v.1.12. (Olympus Optical Co., Hamburg, Germany). The percentage of senescent cells in relation to all cells was counted for each field of view.

### 4.6. Optimization of cMSC(WJ)s Culture Conditions

For optimization of the cMSC(WJ)s culture conditions, the following two different factors were tested: (1) the coating of the culture surface with gelatin; (2) supplementation of GM with fibroblast growth factor 2 (FGF2). For coating, a sterile solution of 1% (*w*/*v*) gelatine from porcine skin (Cat# G2500, Sigma, Merck KGaA, Darmstadt, Germany) in dH_2_O was used. A solution was poured on empty cell culture surface, left for 1 min, and discarded. Dishes were dried before cell seeding. FGF2 (human recombinant, cat# F0291) was used at a concentration of 2.5 ng/mL. Both described factors were used alone and in combination resulting in 4 different culture conditions as follows: (1) GM (CTRL), (2) GEL (gelatin), (3) FGF2, (4) GEL_FGF2.

### 4.7. Confirming MSCs Identity of Isolated Cells

Identification was performed after thawing at the fourth passage and included testing the ability for three-lineage differentiation and the evaluation of surface marker expression. For human cells, we followed the requirements defined by the International Society for Cell and Gene Therapy [15], whereas for canine cells, we followed the guidelines proposed by Guest et al. [25]. To ensure that the modified culture conditions did not affect the basic characteristics of canine MSCs, the identification study for canine cells was performed on cells that had been cultured on gelatin with the addition of FGF2 since isolation.

#### 4.7.1. Trilineage Differentiation

Cells from both species (hMSC(WJ)s and cMSC(WJ)s) were tested in their ability to perform adipogenic, osteogenic and chondrogenic differentiation. For adipogenic differentiation, the cells were cultured in DMEM high glucose supplemented with 5% rabbit serum, 100 nM dexamethasone, 200 μM indomethacine, 4 μM insulin, 0.5 mM 3-Isobutyl-1-methylxanthine, and 5 μM rosaglitasone. After 2 weeks, the cells were fixed and stained with Oil Red O (ORO) using a standard protocol. Briefly, after fixation, and washing with 60% isopropanol, cells stained with 0.5% ORO in isopropanol diluted with dH_2_O 6:4, pH 7.5, for 15 min, RT. For osteogenic differentiation, the cells were cultured in DMEM high glucose supplemented with 100 nM dexamethasone, 50 μM L-Ascorbic acid 2-phosphate, and 10 μM β-glycerophosphate. After 2 weeks, cells were stained with 2% (*w*/*v*) solution of Alizarin Red in acidic PBS (pH—4.2) for 10 min in 37 °C. For chondrogenic differentiation, 5 × 10^5^ of cells were suspended in DMEM high glucose supplemented with 100 ug/mL sodium pyruvate, 1% (*v*/*v*) insulin–transferrin–selenium, 10 ng/mL TGFβ2, 100 nM dexamethasone, and 100 μM L-Ascorbic acid 2-phosphate, centrifuged in a conical centrifuge tube and left for the subsequent 2 weeks for the formation of a chondropellet (medium partially replaced every third day). The formed spherical structures were then embedded in paraffin, cut into preparations, and stained with Toluidine blue (0.1% *w*/*v*, pH: 2.5, 10 min, 37 °C. All media supplements and reagents for staining were from Sigma-Aldrich, Saint Louis, MI, USA).

#### 4.7.2. The Assessment of Cell Markers Expression by Flow Cytometry and Immunofluorescence

For human cells, the analyzed profile included CD44, CD73, CD90, CD105, CD11b, CD19, CD34, CD45, and HLA-DR. The cells were stained using the BD Stemflow™ hMSC Analysis Kit (BD Biosciences, San Jose, CA, USA) in accordance with the manufacturer’s instruction. For canine cells, the analyzed profile included CD44, CD90, CD45, and CD11b. Detailed information about antibodies used is presented in Supplementary Table S1.

Stained cells were evaluated on DxFLEX Flow Cytometer (Beckman Coulter, Indianapolis, IN, USA) with Cytexpert software v. 2.5. Beckman Coulter, Indianapolis, IN, USA). For testing vimentin expression using immunocytochemical staining, anti-vimentin antibody was used (1:100, clone V9, Dako, overnight, 4 °C), followed by a secondary fluorochrome-conjugated antibody (Donkey Anti-Mouse antibody, 1 h, RT, 1:300). Cell nuclei were stained with DAPI. Cells were visualized using Cytation™1 automated fluorescent microscope (BioTek, Agilent, Santa Clara, CA, USA).

### 4.8. The Assessment of Lymphocyte Proliferation Using Dye Dilution Assay

#### 4.8.1. Isolation of Peripheral Blood Mononuclear Cells (PBMCs)

Human PBMCs were isolated from the buffy coats of healthy anonymous blood donors. Buffy coats were diluted 1:2 in PBS and, after a density gradient centrifugation with Histopaque-1077 (Sigma-Aldrich, Merck KGaA, Darmstadt, Germany) (20 min, RT), the interphase containing PBMCs was collected, washed, treated with RBC lysis buffer, and then cryopreserved. Canine PBMCs were isolated from the whole blood of healthy anonymous blood donors from commercial blood bank for veterinary patients. The blood was diluted 1:2 in sterile PBS and layered on a density gradient medium for the isolation of mononuclear cells (Lymphoprep, Stemcell Technologies, Vanouver, BC, Canada). Centrifugation was performed in SepMate PBMC isolation tubes (Stemcell Technologies) at 800× *g*, 10 min, RT. Then, the isolated cells were washed twice with PBS supplemented with 2mM EDTA and 2% FBS. Red blood cells were removed using ACK lysing buffer (Thermo Fisher Scientific, Waltham, MN, USA), and then the PBMCs were cryopreserved in FBS/10% DMSO (Sigma-Aldrich, Merck KGaA, Darmstadt, Germany).

#### 4.8.2. Co-Culture of MSCs and PBMCs

Co-culture experiments were always conducted in a species-compatible manner—human MSCs were cultured with human PBMCs and canine MSCs with canine PBMCs. In each case, the MSC and PBMC donors were unrelated to each other.

MSCs were seeded on a 96-well plate at a density of 2 × 10^4^/well. PBMCs were thawed at the same day and seeded on a 6-well plate at a density of 10 × 10^6^/well in medium for lymphocytes (MLym), consisting of RPMI-1640 GlutaMAX™ supplemented with 0.1% HEPES, 10% FBS, 1% Sodium pyruvate, 1% nonessential amino acids, 1% penicillin, and streptomycin; all from Gibco, Thermo Fisher Scientific, Waltham, MN, USA). Both MSCs and PBMCs were left overnight. The next day, unattached PBMCs were collected and stained with proper dye that enables assessment of cell proliferation. Human PBMC were stained with CellTrace™ CFSE Cell Proliferation Kit, and canine PBMCs with CellTrace™ Far Red Cell Proliferation Kit (both from Invitrogen, Thermo Fisher Scientific, Waltham, MN, USA), according to the manufacturer’s instruction. Some CFSE- and Far Red-marked PBMCs were at this stage prepared for flow cytometry analysis (description below), fixed and kept at 4 °C (protected from light) until the end of experiment. These cells constituted the day 0 control. The remaining CFSE- or Far Red-stained PBMCs were suspended in medium MLym and seeded on a 96-well plate at a concentration of 25 × 10^4^/200 µL/well. They were either added to already adhered and washed MSCs (the ratio of PBMCs to MSCs was approximately 10:1) or seeded alone. Half of the wells were left unstimulated, and the other half was stimulated with Concanavalin A (ConA, Thermo Fisher Scientific, Waltham, MN, USA) at a concentration of 5 μg/mL. Therefore, each experimental set consisted of the following variants: unstimulated PBMCs (NS), ConA-stimulated PBMCs (ConA), unstimulated PBMCs with MSCs (NS_MSC), and ConA-stimulated PBMCs with MSCs (ConA_MSC). Each variant was run in triplicate. The cells were incubated for 5 days (5% CO_2_, human MSCs and PBMCs 37 °C/canine MSCs and PBMCs 38.5 °C). At day 3, 50 µL of fresh medium (Mlym) was added to each well. Each PBMC population was co-cultured with MSCs from 2 different donors.

#### 4.8.3. Flow Cytometry Analysis

At the end of the experiment (day 5), non-adherent cells were collected, cells from technical replicates were pooled and prepared for flow cytometry analysis. All samples were washed with FACS buffer (PBS supplemented with 2% FBS) and stained with viability dye from the LIVE/DEAD Fixable Blue Dead Cell Stain Kit (Invitrogen, Thermo Fisher Scientific, Waltham, MN, USA), as well as species-specific antibodies against CD3, CD4, CD8 antigens (20 min, RT). The details for the antibodies used are provided in Supplementary Table S2.

After incubation with the viability dye and specific antibodies, the cells were washed twice with FACS buffer, and then resuspended in 200 uL of FACS buffer. Flow cytometry acquisition was performed on a BD FACS Aria II flow cytometer (Becton Dickinson, Heidelberg, Germany). The proliferation of lymphocytes was analyzed using Proliferation platform from FlowJo software v10 (Ashland, OR, USA) in accordance with the manufacturer’s instruction. The following four proliferation indexes were calculated as described by Roederer [29]: (1) Division Index (total number of divisions/the number of cells at start of culture), (2) Proliferation Index (total number of divisions/cells that went into division), (3) Expansion Index (total number of cells/cells at start of culture), and (4) Replication Index (total number of divided cells/cells that went into division).

### 4.9. Pro-Inflammatory Stimulation of MSCs

For some experiments, the MSCs were stimulated with pro-inflammatory factors. In the preliminary phase, the following three factors were used: IFNγ, TNF, and Poly(I:C). IFNγ and TNF were used in a species-specific manner—human recombinant IFNγ (R&D System, Minneapolis, MN, USA, Cat#: 285-IF) and human recombinant TNF (Peprotech, Cheshire UK, Cat#: 300-01A) were added to hMSC(WJ)s, whereas canine recombinant IFNγ (R&D System, Cat#: 781-CG) and canine recombinant TNF (R&D System, Cat#: 1507-CT) were added to cMSC(WJ)s; all at a concentration of 20 ng/mL. The synthetic ligand for TLR3-Poly(I:C) High Molecular Weight was used at a concentration of 5 µg/mL (InvivoGen, Toulouse, France, Cat#: tlrl-pic, for cells from both species). The concentrations of pro-inflammatory agents were chosen based on previously published data [27,31,34,36]. The agents were used alone and in combination, which resulted in 7 different treatments as follows: (1) IFNγ; (2) TNF; (3) poly(I:C); (4) IFNγ + TNF; (5) IFNγ+ poly(I:C); (6) TNF+ poly(I:C); (7) IFNγ + TNF + poly(I:C). Cells cultured in basic growth medium served as a control. Based on preliminary data, for the final experiments, the following three treatments were chosen: IFNγ, Poly(I:C), and IFNγ+ Poly(I:C). Cells were cultured for 24 and then analyzed for TGFβ secretion (4.10) and IDO1 protein expression (4.11). The same cells cultured without pro-inflammatory factors served as control.

### 4.10. The Secretion of Transforming Growth Factor Beta 1 (TGFβ-1)-ELISA

For the evaluation of TGFβ secretion, MSCs were seeded on a 12-well plate at a density of 6 × 10^4^/well in GM medium. When subconfluence was reached, the cells were washed and exposed to pro-inflammatory stimulation as follows: 20 ng/mL IFNγ, 5 μg/mL Poly(I:C), and IFNγ+ Poly(I:C). The basal medium (720 μL/well) for this experiment was Opti-MEM™ (Cat#31985062, Gibco™) without the addition of serum to exclude serum-derived TGFβ-1 interference with ELISA assay. After 24 h, the supernatant was collected, centrifuged to eliminate cells and cell debris, aliquoted, and then immediately frozen in −80 °C. TGFβ concentration was assessed using the TGF-beta 1 Quantikine ELISA kit (Cat#: DB100C, R&D system), dedicated to both human and canine material. The assay was performed in accordance with the manufacturer’s instruction. The medium alone was used as a control. Cells from six different donors (both human and canine) were used in this analysis. All samples were analyzed in duplicates. Canine serum (*n* = 2) was used as positive control.

### 4.11. The Expression of Indoleamine-2,3-Dioxygenase 1 (IDO1) Using Western Blot

For the Western blot analysis, MSCs were seeded on Primaria™ (ø 100 mm) Petri dishes (1 dish per one treatment condition). Once the cells reached subconfluence, they were exposed to different pro-inflammatory stimulations as described above. The basal medium for this experiment was DMEM supplemented with 10% FBS. After 24 h, cells were washed twice, scraped from the culture surface and centrifuged. Cell pellets were frozen in −80 °C. Cells were lysed and total protein fraction was isolated using RIPA buffer (Sigma) with proteinase and phosphatases inhibitors (Cat# P8340, Cat#P5726). The protein concentration was measured with the use of the Bradford reagent. From each sample, 40 µg of protein was loaded on a polyacrylamide gel and separated in electrical gradient. The proteins were transferred to the PVDF membrane (Immobilon^®^—FL, Cat#IPFL00005, Millipore, Merck KGaA, Darmstadt, Germany). Non-specific binding was prevented by incubation of blots in 5% (*w*/*v*) solution of powdered nonfat milk in TBST (Tris-Buffered Saline with 0.1% Tween^®^ 20 detergent). Next, the membranes were incubated with anti-IDO1 antibody (Rockland, cat# 210-301-E58, 1:1000, overnight, 4 °C). After washing, the secondary antibody was used (anti-mouse IgG conjugated with IRDye^®^ 680, Licor, cat# 926-68072, 1:5000, 1 h, RT). Beta-actin was used as loading control by using anti-βactin antibody (Santa Cruz, cat# sc-47778, 1:1000, overnight, 4 °C) followed by IRDye^®^ 680 (same as above). The membranes were analyzed using ChemiDoc™MP Imaging System (Bio-Rad Laboratories, Inc., Hercules, CA, USA). Optical density of bands was analyzed using ImageLab v. 6.1 software (BioRad Laboratories, Inc., Hercules, CA, USA).

### 4.12. Statistical Analysis

All the statistical analyses were performed using STATISTICA v. 13.1 software (StatSoft^®^, Hamburg, Germany). In each analysis, the first step was the assessment of data distribution within groups using Shapiro–Wilk test. If data were compared to the control, the groups of related data with abnormal distribution were analyzed using the Wilcoxon matched-pairs signed-rank test, and the groups with confirmed normal distribution were compared using Student’s *t*-test. If two groups of unrelated data were compared to each other, the groups with abnormal distribution were analyzed using the Mann–Whitney U test and the groups with confirmed normal distribution were compared using Student’s *t*-test. Analyses of multiple groups (i.e., comparing groups during optimization test) were performed using ANOVA with Tukey’s post hoc tests. Significance was set at *p* < 0.05 and graphs were presented as mean ± standard error of the mean (SEM) if not stated otherwise.

## Data Availability

Data are contained within the article or Supplementary Materials.

## Supplementary Materials

The following supporting information can be downloaded at: https://www.mdpi.com/article/10.3390/ijms25168926/s1. Reference [43] are cited in the supplementary materials.
