# Supplementary material for: The Comparison of Immunomodulatory Properties of Canine and Human Wharton Jelly-Derived Mesenchymal Stromal Cells
Source: Int J Mol Sci. 2024 Aug 16;25(16):8926. doi: 10.3390/ijms25168926 (PMC11354339; doi:10.3390/ijms25168926)
Supplement: Supplementary file 1 [file ijms-25-08926-s001.zip › ijms-3140354-supplementary.pdf]

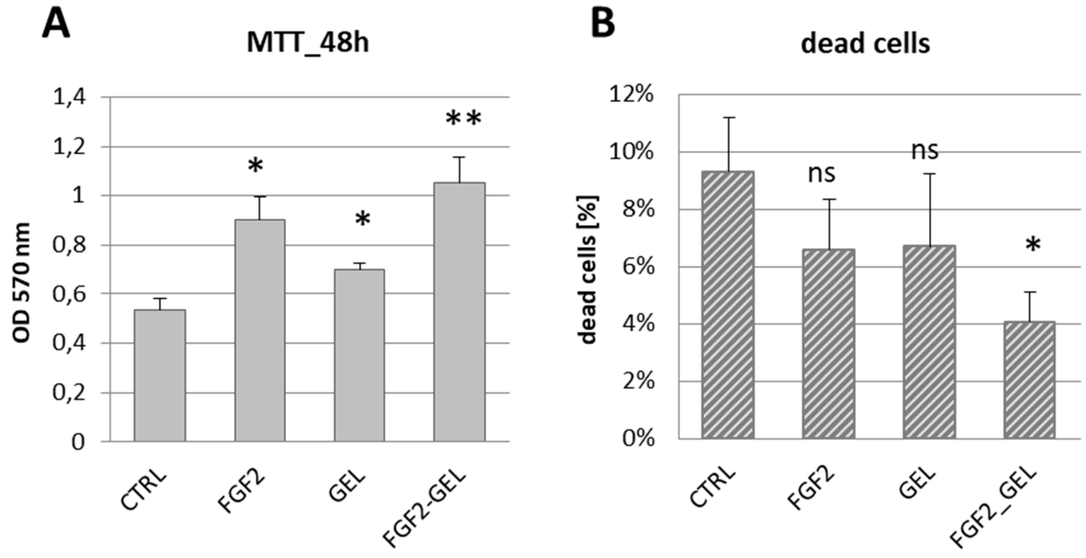

Supplementary Figure S1. The effect of different culture conditions on viability and proliferation of canine umbilical cord-derived cells. A) MTT assay, B) dead cell proportion assessment based on Trypan blue staining. FGF2 - cells cultured on uncoated surface in growth medium (GM) supplemented with 2.5ng/mL fibroblast growth factor 2, GEL – cells cultured in GM on a surface coated with porcine gelatine, FGF2_GEL – cells cultured on gelatine coated dishes in GM with FGF2; ns – statistically not significant, *, p<0.05; **,p<0.01 in comparison to control (using Wilcoxon test or t-Student for related data test depending on data distribution), n=6-7.

Supplementary Table S1. List of reagents used for identification of canine UC-derived cells.

	**Clone**	**Fluorochrome**	**Cat no**	**Antibody specificity**
CD44	IM-7	PE	#12-0441-82	used in canine cells [1,2]
CD90	YKIX337.217	APC	#17-5900-42	Anti dog
CD45	YKIX716.13	FITC	#11-5450-41	Anti dog
CD11b	M1/70	V450	#560456	used in canine cells [3]

Human MSCs were identified using a ready set of antibodies from kit: BD Stemflow™ hMSC Analysis Kit.

Supplementary Table S2. List of reagents used for analysis of lymphocytes proliferation in both human and canine samples.

**Cell origin**	**antigen**	**Clone**	**Fluorochrome**	**Cat no**
**human**	CD3	SK7	PerCP	#557851
	CD4	SK3	APC Cy7	#341115
	CD8	SK1	APC	#345775
	Viability	NA	V450	#L23105
	proliferation	NA	FITC (CFSE)	#C34570
**canine**	CD3	CA17.2A12	FITC	#MCA1774F
	CD4	YKIX302.9	PE-Cy7	#17-5040-42
	CD8	YCATE55.9	PE	#MCA1039PE
	Viability	NA	V450	#L23105
	proliferation	NA	APC	#C34564

NA- not applicable

References:

1. Wright, A.; Snyder, L.; Knights, K.; He, H.; Springer, N.L.; Lillich, J.; Weiss, M.L. A Protocol for the Isolation, Culture, and Cryopreservation of Umbilical Cord-Derived Canine Mesenchymal Stromal Cells: Role of Cell Attachment in Long-Term Maintenance. *Stem Cells Dev.* **2020**, *29*, 695–713, doi:10.1089/scd.2019.0145.
2. Saulnier, N.; Loriau, J.; Febre, M.; Robert, C.; Rakic, R.; Bonte, T.; Buff, S.; Maddens, S. Canine Placenta: A Promising Potential Source of Highly Proliferative and Immunomodulatory Mesenchymal Stromal Cells? *Vet. Immunol. Immunopathol.* **2016**, *171*, 47–55, doi:10.1016/j.vetimm.2016.02.005.
3. Jackson, K.; Milner, R.J.; Doty, A.; Hutchison, S.; Cortes-Hinojosa, G.; Riva, A.; Sahay, B.; Lejeune, A.; Bechtel, S. Analysis of Canine Myeloid-Derived Suppressor Cells (MDSCs) Utilizing Fluorescence-Activated Cell Sorting, RNA Protection Mediums to Yield Quality RNA for Single-Cell RNA Sequencing. *Vet. Immunol. Immunopathol.* **2021**, *231*, 110144, doi:10.1016/j.vetimm.2020.110144.
